# The Pathogenesis of Epilepsy

**Published:** 1889-08

**Authors:** Arthur E. Mink

**Affiliations:** 54 Park Avenue; Rochester, N. Y.


### *THE PATHOGENESIS OF EPILEPSY.*[1]

By ARTHUR E. MINK, M. D., Rochester, N. Y.

There is no disease of more interest, and none which has afforded more food for speculation, than epilepsy. Pathological anatomy has been ransacked to furnish for it a seat and a cause, and so high an authority as Esquirol considered the cartilaginous plates which he found in the arachnoid of some epileptics as the anatomical cause of the disease. Irregularities are sometimes found in the crania of epileptics. The crania are sometimes too large, and sometimes too small, and in some cases the skulls show a more or less prominent asymmetry. Behrend found the occiput of epileptic children to be considerably flattened. Müller, among forty-three epileptics, found normal crania only three times, while Rieken has shown that the right half of the skull remains in a lower state of development than

1. Read at the annual meeting of the Monroe County Medical Society, May 29, 1889.

the left. Hoffman states that just the opposite is the case. In later times, Benedikt has made some cephalometric researches on the crania of epileptics, and has found that, in 120 epileptics, 70.8 per cent. show an atypical condition of the skull. As regards the pathological conditions found in the interior of the cranium, there are the same conflicting statements. Hardly any two observers agree. Follet asserts that the two cerebral hemispheres show an important difference in weight, and he is confirmed in this by Bucknill and Echeverria. In some cases, stenosis of the foramen magnum has been found, and Nothnagel asserts that this is well calculated to cause, by pressure or other influences, an attack of epilepsy. Lesions of the meninges, such as thickenings, adhesions, and exudates, are among some of the most frequent conditions found in epilepsy. Lately, Meynert has confirmed the dissections of older authors regarding the presence of atrophy and sclerosis of the cornu ammonis, but it is by no means a constant lesion, and Bourneville has found many cases without it. So much for the pathological anatomy of epilepsy, and for the conflicting statements found therein. For us, all of this discord is sufficient to convince us of this one fact, viz.: That epilepsy is not an individual disease, but only a symptom, of which the various pathological conditions which have been found can each be a cause under various circumstances. They act by being what Hughlings Jackson calls "discharging lesions," and that is by setting up in the grey matter of the brain a state of abnormal irritability. A very slight stimulus then suffices to liberate a sudden discharge of nervous energy. In the ordinary state, the protoplasm of the nerve-cells is in a condition of unstable equilibrium, and under the excitus of normal stimuli it liberates its energy in the form of moderate and coördinated nerve discharges. Now, the various pathological conditions act as a cause of epilepsy, by inducing a condition of hypermetrition in the nerve-cells of the motor centers; so that only the slightest stimulus is needed to liberate and cause that sudden discharge of nervous energy known as an epileptic attack. We hold that, in the light of modern cerebral pathology and physiology, the old distinctions between idiopathic and traumatic epilepsy must be done away with. Now, the state of morbid irritability of the cortex can be brought about by various pathological processes, and we can define epilepsy as a sudden discharge of nervous force from the grey matter of the brain. But it is here that the doctrine of cerebral localization, as in so many parts of cerebral pathology, has been of such immense importance, although, long before, Hughlings Jackson had anticipated some of its most important facts on clinical grounds alone. He also made a great advance by his distinction between destroying and discharging lesions. It was not until 1872, however, that Hitzig gave experimental proof of these ideas, by not only showing that electrical stimulation of various portions of the brain called forth various movements, but also that pathological processes of various kinds set up in the cerebral motor centers were capable of producing so-called idiopathic epilepsy. Ferrier next showed that strong electrical stimulation of the motor centers produced, first, unilateral convulsions, or what is now called hemispasms or Jacksonian epilepsy. He found that these rapidly became general convulsions.

These facts and experiments have all been confirmed by various observers. Eulenburg and Landois have produced epileptiform convulsions in dogs by mere chemical irritation of the motor centers. Albertoni has produced the same by electrical stimulation of a circumscribed area near the crucial sulcus, and Kolman-Balogh speaks of no less than eight centers, the strong stimulation of which produces general convulsions. Luciani has made some interesting experiments in regard to dogs made epileptic by various injuries done to the cerebral cortex. He has found that when he extirpated the motor centers, that those parts which are supplied by these remained free from convulsions. Indeed, after a short time, he states, the animals regained the normal control of all their muscles.

Pitres and Franck extirpated, in the same manner, the motor centers from dogs, and found that in a short time the cortex in the neighborhood of these centers became inflamed, and that only a slight stimulus would cause epileptiform convulsions, although the parts whose centers were extirpated remained free from convulsions.

There has been, of course, hardly any opportunity to verify these facts in an experimental way upon the human subject, and the only one who has had the opportunity and the boldness to do so was our own Roberts Bartholow. He had a woman whose brain had been laid bare by cancerous ulceration. He stimulated the motor region, and produced general convulsions; but, a short time after, the woman died. All of these experiments are conclusive evidence, to our mind, that epilepsy is a symptomatic phenomenon, caused by various lesions which act as discharging lesions, and thereby produce a sudden explosion of nerve energy.

As we have said before, there are many pathological conditions which can produce epilepsy. Cerebral trauma, cerebral anemia, syphilis in all of its various forms in which it affects the brain, can each in turn become a cause of epilepsy.

Among the various contributing causes to an epileptic attack is cerebral anemia.

Long ago, it was known that animals bled to death exhibited epileptic convulsions, and Travers and Marshall Hall believed it to be the chief cause of epilepsy. Astley Cooper tied the carotids of animals and produced epilepsy, and recently Kussmaul and Tanner have shown how important a factor is cerebral anemia.

Nothnagel has shown that there are secondary spasm centers in the pons and medulla, and the researches of Schroeder Vander Kolk on the medulla oblongata of epileptics, is yet a classic. He found strong evidences of hyperemia of the fourth ventricle, ecchymoses at the nuclei of the vagus and hypoglossus, and dilatation of the vessels in the substance of the medulla and about the olives. But these observations in no way conflict with the discharge theory,—in fact, the yare in perfect consonance with it.

For the primary discharges from the cerebral cortex, in passing down the centrifugal cerebral tracts, thus excite the secondary spasm centers in the pons and medulla, and these then assist in causing the epileptic attack.

54 Park Avenue.

